# Meaning Making Process and Recovery Journeys Explored Through Songwriting in Early Neurorehabilitation: Exploring the Perspectives of Participants of Their Self-Composed Songs Through the Interpretative Phenomenological Analysis

**DOI:** 10.3389/fpsyg.2018.01422

**Published:** 2018-08-07

**Authors:** Felicity A. Baker, Jeanette Tamplin, Nikki Rickard, Peter New, Jennie Ponsford, Chantal Roddy, Young-Eun C. Lee

**Affiliations:** ^1^Faculty of Fine Arts and Music, The University of Melbourne, Melbourne, VIC, Australia; ^2^Psychological Sciences, Monash University, Melbourne, VIC, Australia; ^3^Spinal Rehabilitation Service, Caulfield Hospital, Alfred Health, Melbourne, VIC, Australia; ^4^Rehabilitation and Aged Services, Medicine Program, Monash Health, Melbourne, VIC, Australia; ^5^Epworth-Monash Rehabilitation Medicine Unit, Monash University, Melbourne, VIC, Australia; ^6^Department of Epidemiology and Preventive Medicine, Monash University, Melbourne, VIC, Australia

**Keywords:** songwriting, music therapy, identity, recovery journey, spinal cord injuries, acquired brain injury, selfconcept, meaning making

## Abstract

**Objectives:** This pilot study examined how 15 participants in early rehabilitation described their self-composed Songs 6- to 12-months following participation in a 6-week identity-focused songwriting program. Specific focus was given to the process of meaning making and identity reconstruction in the participants’ self-composed songs.

**Methods:** Data were collected through individual semi-structured interviews (*n* = 15) and analyzed using interpretative phenomenological analysis. Findings were developed idiographically as super-ordinate themes unique to each participant, then analyzed across cases to identify recurrent themes and subthemes.

**Results:** Participants described the songwriting process as taking them through one of four distinct recovery journeys described by individuals following acquired neurodisability who underwent a focused therapeutic songwriting program. These included (1) re-conceptualizing values and shifting perspectives about self (my body is broken but my mind has been set free); (2) recognizing acquired inner resources to negotiate discrepancies in self (hope is there); (3) confirming existing values and identifying resources and coping strategies (I have what I need to move forward); (4) confirming previously held values and ongoing process of negotiating discrepancies in self (I don’t yet have the answers).

**Conclusion:** The current study provides insight into the nature and process of meaning making and recovery journeys perceived by individuals with neurodisability. Our findings suggest that songwriting could be a therapeutic tool to facilitate identity reconstruction in neurorehabilitation.

## Introduction

Self-identity or self-concept is a broad term used to describe a set of characteristics that we perceive as our own and that are enduring, continuously evolving over time, and are shaped by our experiences and social interactions (Ownsworth, 2014). When people acquire a neurological disability, they can struggle to process numerous physical, cognitive and/or emotional changes, which may threaten the known self (Carroll and Coetzer, 2011). Such threats to the known self may lead to an internal conflict and to disturbed psychological equilibrium (Cicerone et al., 2004; Mateer et al., 2005). When internal struggles occur, these may further impact people’s mental health and ability to function, including successful reintegration back into the community, returning to previous roles, and their capacity to maintain relationships (Ownsworth and Gracey, 2011).

Using a quantitative measure of self-concept, Tyerman and Humphrey (1984) found discrepancies between past and future self-ratings, as well as “striking similarity” between pre-injury and future self-ratings in individuals following severe traumatic brain injury (TBI). These findings have been explained in terms of a discrepancy between pre-injury and current selves and the prospect of resuming pre-injury roles in the face of sustained impairments. These discrepancies exacerbate the identity disturbance. Over time, heightened distress associated with persisting negative self-discrepancies may produce a sense of hopelessness and lead to maladaptive coping and disengagement from rehabilitation and from society (Ellis-Hill and Horn, 2000; Doering et al., 2011).

Individuals who actively pursue continuity in their identity (i.e., “It’s the same me”) as a process of re-establishing one’s sense of self and place in the world, are more likely to experience positive long-term adjustment (Carroll and Coetzer, 2011; Wolfenden and Grace, 2012; Gendreau and de la Sablonnière, 2014). According to Ylvisaker and Feeney (2000), identity continuity is often achieved through re-connecting with one’s values, activities, social networks and roles (e.g., parent) while confronting functional impairments and limitations (e.g., inability to drive). As such, successful identity reconstruction ultimately involves individuals exploring, and revising their self-concept, adjusting to a change in various aspects of self, and modifying future goals (Ellis-Hill et al., 2008).

There is a growing body of research demonstrating that individuals who experience life-altering events, such as an acquired brain injury (ABI), can experience positive psychological outcomes or ‘post-traumatic growth’ (Rogan et al., 2013; Zeligman et al., 2018). Post-traumatic growth has been conceptualized as a process by which individuals find new meaning and use the injury/illness as the opportunity to re-evaluate core priorities and anticipated goals (Hawley and Joseph, 2008). The presence of meaning and social support have been implicated as the strongest predictors of post-traumatic growth in individuals living with chronic illnesses (Zeligman et al., 2018). A recent study in a TBI group showed an association between living according to one’s values and improved functional outcomes (Pais et al., 2017).

Therapeutic songwriting is a music therapy method, which is defined as the process of creating, notating, and/or recording music within a therapeutic relationship to address psychosocial, cognitive, psychological and communication needs of the client (Wigram and Baker, 2005; Baker, 2015). Therapeutic songwriting has been utilized by clinicians worldwide in both non-clinical and clinical populations across the lifespan (Baker et al., 2008). Using a previously developed therapeutic songwriting protocol (Tamplin et al., 2016) designed to promote reconstruction of self-concept in individuals with acquired neurological injuries, we analyzed the song lyrics of the three songs that each participant wrote (Baker et al., 2017). The songs focused on exploring self-concept using six domains (personal self, academic self, moral self, family self, social self, and physical self). The three songs focused on describing their self-perceptions of their pre-injured self (Song 1), present self (Song 2), and imagined future self (Song 3). An independent deductive analyses of 36 songs composed by 12 adults with spinal cord injury (SCI) and 11 adults with ABI showed that individuals tended to focus predominantly on the family and personal self when reflecting on who they were pre-injury while the songs moved toward a focus on the physical self when examining their present self. It was found that the song about their future self began to move toward a more balanced self-concept with many of the domains of the self-explored in detail.

Additional studies using the songwriting protocol with people with SCI (Roddy et al., 2017) and ABI (Roddy et al., 2018) indicated that there were some participants whose self-concept and well-being indices improved, while those participants with a more significant impairment, did not always demonstrate improvement. These later studies suggest that drilling down into participants’ individualized recovery journeys may help to build a more complex and rich picture of how people with acquired neurological disability utilize a songwriting process tailored specifically to address the self-concept post-injury.

Our research methods used a phenomenological inquiry in that we sought to understand the phenomenon of identity reconstruction in people who were recovering from a SCI or ABI. The phenomenon of interest was how the participants perceived changes in self-concept post-injury and what the impact of a tailored therapeutic songwriting intervention was on that process. Phenomenological inquiry typically explores people’s experiences by analyzing first-person accounts of how they experienced an event (Smith et al., 2009). Unlike the majority of interpretative phenomenological analysis (IPA) studies which focus on analyzing interviews with participants (Smith et al., 2009), our study combined the analysis of songs created during the songwriting process in concert with participants’ own reflections of the song when they re-listened to their songs at a later point in time. In music therapy, novel approaches to understanding a phenomenon using IPA have included video analysis (Lee and McFerran, 2015) and analysis of musical improvisations (Pothoulaki et al., 2012). The IPA approach we undertook was guided by IPA principles of phenomenology (the study of the lived experience), hermeneutics (multiple interpretative processes), and idiography (understanding the unique and often subjective phenomenon) (Smith et al., 2009).

Our study aimed to gain an understanding of:

1. What participants described as key messages in their self-composed songs;
2. What meanings participants perceived across their self-composed songs.

## Materials and Methods

### Participants

We analyzed data from 15 individuals (male *n* = 11; female *n* = 4) with ABI or SCI (mean age = 48 years; range = 20–66 years) who were undergoing inpatient rehabilitation or discharged from a sub-acute rehabilitation center in metropolitan Melbourne, VIC, Australia. Pseudonyms were allocated to each participant to protect their identity. Participants were an average of 300 days post-injury (*SD* = 275 days). The nature of injury or illness included ABI (i.e., stroke, TBI, Guillain Barre syndrome), traumatic SCI, and non-traumatic SCI (i.e., spinal stenosis, arteriovenous malformation), as outlined in **Table 1** below.

**Table 1:** Participant demographic and clinical characteristics.

Participant	Gender	Age	Education	Marital status	Injury description
Melanie	F	20	Completed High School/V.C.E.	Single	SCI – post-MVA
Peter	M	50	Postgraduate University Degree	Married/defacto	SCI – sporting accident
Sam	M	44	Completed High School/V.C.E.	Married/defacto	SCI – post-MVA
James	M	64	Completed High School/V.C.E.	Married/defacto	ABI – Guillain Barre syndrome
Valerie	F	27	Postgraduate University Degree	Single	Non-traumatic SCI – arteriovenous malformation
Tony	M	61	Undergraduate University Degree/Graduate Diploma	Single	ABI – Guillain Barre syndrome
Hayley	F	37	No higher than Year 10 of high school	In a relationship but not living together	ABI – subarachnoid hemorrhage
Kelly	F	37	Completed apprenticeship/TAFE/College Diploma	In a relationship	Non-traumatic SCI – lumbar spine canal stenosis
Billy	M	29	Completed apprenticeship/TAFE/College Diploma	Single	SCI – post-MVA
Max	M	60	Completed apprenticeship/TAFE/College Diploma	Divorced/separated	SCI – post-bicycle accident
Richard	M	51	Undergraduate University Degree/Graduate Diploma	Married/defacto	ABI – left middle cerebral artery infarct
Tom	M	66	Undergraduate University Degree/Graduate Diploma	Married/defacto	SCI – post-fall
Finn	M	57	Undergraduate University Degree/Graduate Diploma	Married	ABI – Guillain Barre syndrome
Matthew	M	64	No higher than Year 10 of high school	Married	ABI – multifocal strokes
David	M	46	No higher than Year 10 of high school	In a relationship but not married	SCI – central cord syndrome

*ABI, acquired brain injury; MVA, motor vehicle accident; SCI, spinal cord injury*.

The project received ethical approval from the Austin Health Human Research Ethics Committee (HREC REF H2013/05038). All participants gave written informed consent prior to participating in the study.

### Procedure

A detailed description of the 6-week identity-focused songwriting intervention with the theoretical framework underpinning the intervention has been detailed elsewhere (Tamplin et al., 2016). In summary, participants in the current study created three songs; Song 1 about their past self, Song 2 about their present self, and Song 3 about their imagined future self. A qualified music therapist facilitated the songwriting protocol by assisting the participants to identify key aspects of themselves according to the previously described subdomains of the self-concept and to assist the participants to shape these into meaningful lyrics and create accompanying music.

### Data Collection

Individual semi-structured interviews were conducted 6 months post-completion of the intervention and participants were encouraged to reflect on their experience of songwriting. The interviews allowed for sensitivity, reflexivity and flexibility for ‘participants to think, speak and be heard’ (Reid et al., 2005, p. 22). Interviews were conducted by either YL or CR. Interviews were 40–50 min in length. Questions posed to the participants were formulated to elicit the participants’ listening experience and perspectives on their songs at 6-month follow-up. After participants listened to each song, they were asked the following questions about each song:

1. What did you mean by the title of this song?
2. What is this song about?
3. Can you tell me about what is the overall message or themes of the song that you were trying to convey?
4. What did you mean by the lyrics in Verse 1, Verse 2, and the Chorus?
5. What were you thinking about when you wrote this song?
6. How does it feel to listen to this song now? Do you think the feelings have changed from when you wrote them?
7. Does this song still have meaning for you now? Why/why not?

### Data Analysis

We transcribed all the interviews and imported these transcriptions into MAXQDA qualitative analysis software. This MAXQDA file was then duplicated so that authors YL and FB could independently read through the interviews and code what participants said about each of the three songs they created. Drawing on hermeneutics, we returned to the interviews on multiple occasions over 6 months, creating themes that reflected participants’ perceptions of the songs’ meaning in the context of their recovery journey. Where appropriate, *in vivo* coding was used to ensure the participants’ intended meaning was not lost during the analysis process. YL and FB, then independently distilled all the codes to arrive at an interpretation of the meaning behind each song and the story of recovery that the song was communicating. Following this, YL and FB compared their analysis of each song, and arrived at similar distillations, sometimes differing in terminology when aggregating several codes. Examples of these can be reviewed in **Table 2**.

**Table 2:** Examples of how author 1 and author 2 distilled their analysis into the essence of each song.

	Song 1 message/theme (FB)	Song 1 message/theme (YL)	Song 2 message/theme (FB)	Song 2 message/theme (YL)	Song 3 message theme (FB)	Song 3 message/theme (YL)
Matthew	I never gave up despite obstacles; describe life, pain, but still remaining positive	Remaining positive in the face of challenging past life (health)	Capturing difficult period in hospital	Capturing dark period in hospital	Tragedy makes you review your life	Importance of family to get him through the challenges of stroke
Kelly	Reflection about self-worth, loving herself and letting go of the past	Self-worth, allowing self to be loved again; song expressing unresolved feelings re: marriage breakdown	Living in a new body (adjusting to physical changes/from being an athlete); holding onto hope	Living in a new body, hope is healing; message to self (like a mantra to get her through difficult times)	Accept aids and accept myself; positive messages of affirmation; hope is there	Setting realistic expectations for the future; balance between hope and realistic expectations

Following this process, FB and YL compared the distilled stories across cases, and looked for common threads. After immersing themselves in the data for an extended period of time, they grouped participants’ stories according to similarity in journeys through the songwriting process and then aggregated the stories from each group to arrive at composite journeys.

## Results

**FIGURE 1:**
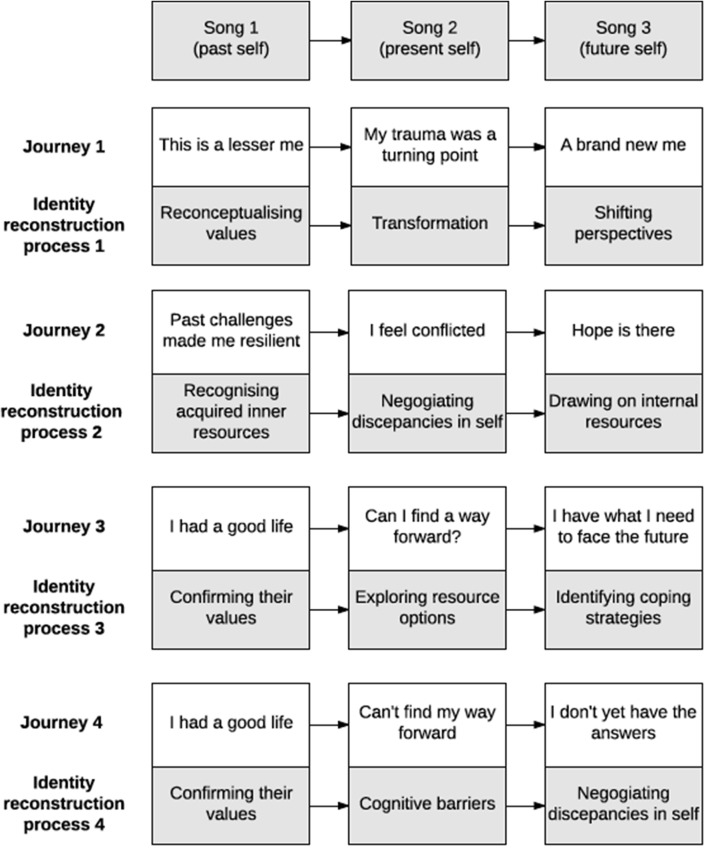
Four recovery journeys and their associated cognitive process of identity reconstruction.

We distilled individuals’ experiences into four distinct journeys, In summary, these were: (1) re-conceptualizing values and shifting perspectives about self (‘my body is broken but my mind has been set free’); (2) recognized acquired inner resources to negotiate discrepancies in self (‘hope is there’); (3) confirming existing values and identifying resources and coping strategies (I have what I need to move forward); and (4) confirming previously held values and ongoing process of negotiating discrepancies in self (I don’t yet have the answers). The four distinct journeys are depicted in **Figure 1** and described in further detail in the subsections below.

### Recovery Journey 1: ‘My Body Is Broken but My Mind Has Been Set Free’

Three participants (Melanie, Peter, and Sam) described a similar journey in their self-composed songs of how their injury/illness provided them with an opportunity to reconceptualise their values and shift their perspectives about self. Melanie described her first song as the process of reconceptualising her values pre-injury, specifically how her injury made her recognize how selfish she was before the injury stating:

“…like looking back at what I – the person that I was… I was there, but I didn’t really… I think I was very, quite selfish…I didn’t appreciate the things I took for granted, like walking and things like that, I just didn’t even give it a second thought, which no-one ever does.”

Similarly, Peter stated that his first song contained key messages about the regret about his lack of purpose and “disappointing” pre-injury life:

“Just take away very proud childhood to a rather disappointing ending really. To that point, I really hope I could have achieved a bit more but initially was really full of hope and then it turned to something else. So, it’s a bit of a disappointing ending.”

In the same vein, Sam described the key messages of his first song to be a reflection about his upbringing and regret about not appreciating his family – “I thought that I wasn’t showing enough affection to her [wife]… with the kids that they mean everything.”

Participants in this journey described Song 2 and Song 3 to reflect how their trauma served as a *turning point* for shifting their perspectives about self and life. Melanie described how her injury was “the best thing that could have happened to me.” She stated that her songs convey the losses and challenges associated with her injury including the “dark” period in hospital and reflects on how the injury has “changed me for the better.” She stated, “now I see the me I was meant to be. It’s like now I get it. This is what I’m supposed to be. It feels right” and added “my body is broken, but my mind has been set free.”

ciated with his injury and how t Similar themes emerged throughout the songs of Peter who stated that his Song 2 and Song 3 reflect on his losses and challenges associated with his injury and how the injury shifted his perspective about self and life:

“All those questions… could I walk… if that won’t happen, will that be the end of me… What if I couldn’t walk, never walk. Then, well miracles do happen. I could walk again. Everything back to normal. Family rejoice. Then the last paragraph [of the song] is it doesn’t matter. Whatever happens, I’ve gone through this journey. Compare it to, I’m lucky I’m alive. Literally second life really. So, it’s whatever happens that’s the brand new me…I look at life very differently now…Even if I go back, it won’t be me anymore. It wouldn’t be the same me because I have more experience… Things have to change for the better.”

Sam described his Song 3 as capturing how his injury/illness redefined his identity and outlook in life – “New and different me…my outlook is not going to change. Not starting over, but just start a new chapter.” Similar to Melanie and Peter, Sam said that Song 3 contained themes about shifting his perspective about himself:

“So, that’s breaking free mentally. No more pleasing people or if someone said something nasty, I’ll just put them back into place. If they say something nice, then I’ll be happy and friendly with them. Everything straight out, straight down the line… my body is still broken but I’m getting better and better.”

### Recovery Journey 2: ‘Hope Is There’

Five out of fifteen participants (James, Valerie, Tony, Hayley, Kelly) described how their resilience acquired through past challenges allowed them to draw on their internal resources in the face of their injury/illness. Specifically, participants described how their self-composed songs explored the process of recognizing their inner resilience from past painful experiences to reconciling the negative discrepancies in self to overcome their current injury/illness.

Three participants (Valerie, Tony, Hayley) stated that Song 1 depicted their challenging early life experiences. Valerie stated that:

“I think I’ve struggled a lot during my early years. This song really expresses who I was before… and then my struggles, to be accepted by the family… Because I think they didn’t really intend to make me feel that way. But unconsciously they were able to or maybe sometimes it’s because – it’s just me. It’s just me because I just keep on thinking that maybe I wasn’t good enough.”

Valerie described how Song 1 captures what she had learnt from her early challenges:

“... the chorus is more telling you that you could be beyond actually what you think yourself could do. Sometimes you just stop listening to what others are telling you. It’s more of listening to yourself because that’s who you are. Then it’s you that makes you you… Don’t forget about the struggles that you had before because that would help you now.”

Two participants (James, Kelly) reflected on a particularly challenging period in life prior to injury/illness in Song 1. For instance, Kelly described how her first song reflected on how challenging past experiences allowed her to cope with the current illness:

“[when I was writing the song] I was thinking about self-worth and that came up a lot when – in rehabilitation and I’ve struggled with self-worth for a big part of my life, particularly during my marriage… then the marriage breaking…So, whether the breakdown actually prepared me mentally for the surgery and the spinal cord injury, I don’t know. So, I think that’s why a lot of that was coming out in that song.”

Valerie described Song 2 as her inner struggle about facing the losses and challenges and the process of negotiating the conflicting identities following her injury:

“Because that moment when I was writing this song, there were so many things playing in my mind. The different aspects of my life that I am thinking that I’m losing them. I’m losing my life. I’m losing my work. I’m losing everything. Of course, you have that eagerness in yourself that I want them back. I want the things that I’ve worked hard for to still be there… I lost one big part of my life… my health… I lost it, because I didn’t value it much.”

Similar themes of negotiating discrepancies in self and recognition of inner resilience from past experience were described by James in Songs 2 and 3.

“Life was good and I didn’t want to lose it. For a change, I had a good life and with kids around and good family life and I just didn’t want to lose that basically…I’m talking about how I beat the cancer and I will beat this one too. I’m getting there so it’s not a problem. If I’m going to stay this way that’s fine. I’m accepting that I’m not 100% but who cares?”

Tony described his emotional conflicts associated with the losses he had experienced due to his illness and the process of negotiating these losses in Song 2:

“Well, basically, I was saying that it’s basically my life in music has died…because of the state of the industry and what I concentrate on and what I was good at, no longer existed. The depression of being sick and the way it makes you feel, all the different emotions go through and that and with so many of my friends dying over the last few years.”

“But it’s the same old story. Bugger it. Don’t let it get you down. Just keep on going. Try something else, try whatever… So, basically, it was a case of get up, keep doing things. Keep doing stuff, if no more than just to show people that you are not crushed by the fact you have got nothing to do anymore.”

Hayley described her Songs 2 and 3 as the process of recognizing and drawing on her existing internal resources to negotiate discrepancies in self:

“I guess even though things might be bad underneath, it’s still – I’m still able to just let it go, concentrate on what has to be done and keep smiling on the outside. Keep being happy and positive. I think that’s the whole positive part, because that’s – positive thinking does really get you everywhere.”

Similarly, Valerie and Kelly stated that Song 3 reflected on the process of drawing on their previously acquired inner resources. Valerie described Song 3 as:

“… this song I said, just- I want to say to myself that everything is going to be alright. Because in the situation I really can’t do so much. I don’t want to force myself again and then compromise or jeopardize my health again because of the things that I wanted. So, it’s like okay everything is going to be alright… It [song] still has a very good mantra that’s telling you to don’t be stressed and just I think trust that everything will fall into place.”

Kelly talked about a similar theme in her Song 3:

“There was hope in there, love myself in there, accept aids and accept myself and don’t think too far ahead. Live for now.”

### Recovery Journey 3: I Have What I Need to Move Forward

Six out of fifteen participants (Billy, Max, Richard, Tom, Finn, Matthew) interviewed described how their songs reflect important values (family, religion, home), exploring internal and external resource options and identifying coping strategies to overcome injury/illness. Billy discussed the importance of reflecting on his past and confirming his values in Song 1:

“Dancing in the past… it’s like, you know, don’t rush through reflection. If you’re looking at your past, don’t rush through it, don’t just skip over it, don’t just… take your time, but don’t just take your time with it. The dancing part is like enjoy, enjoy the highlights of the past.”

“That (song)… I guess this caps off what it means for me to dance through my past, to look back and see how even the sad stuff and the kind of crap I had in my life was turned around because I was allowed to help other people because of it. Because of the experience I’m able to use that experience of good instead of generally sweeping over the past.”

In Song 1, Finn voiced about the start of his new life in Australia as an important period in defining his values pre-injury:

“So yeah, it was taking a step into something really new. It was exciting but it was scary as well… it was all exciting coming to Australia and that’s what I tried to put in the song. Yeah, that’s just the story of how things did happen.

Similarly, Tom reflected on his important values pre-injury in Song 1:

“It’s really about the idea that… you make great progress if you take your starting point from people who have already achieved great things…that you can take inspiration from other people, and you can be stronger and bolder, when you take that inspiration.”

Max described how his first song captured the importance of home and spirituality in defining his values in his pre-injury period:

“It captures my life at home. I thought of my life at home – my neighbor – playing games in the backyard with my kids. The park – when I took my kids to the park…. All the time- take courage to the fact that there’s someone listening to me and worship is pretty much part of that. So, it’s devotion to a listening spirit.”

Tom reflected on the process of exploring the options and resources to establish a new way of living with his acquired injury in Song 2:

“It depends on your perspective, and how you look at things. You can look at the opportunities… what I’m really saying is that I’ve now established a new way of living, and making the most of what I can, and being creatively restless, and not dwelling or feeling sorry for myself…That, I’ve pulled everything back together again, and I’m doing things which I feel are useful and giving me satisfaction.”

In Song 3, Tom described the process of identifying coping strategies and drawing on existing inner strengths to move forward with living with his injury:

“The first step in all of this is to... get all that in place, and then to move forward from there. Well, really, the emphasis is on thrive, that – the emphasis is to make the most of what you now have available… there is no reason why you can’t – why I can make progress and make a valuable contribution to my family, and continue to enjoy life… The key to all of this is to have some hope that particularly technology will – is evolving so fast, that you’ve got to maintain a positive attitude and not fall down. Relax a little bit, and allow things to happen.”

Richard described how Song 3 was about recognizing his values and re-evaluating his goals and priorities, identifying resources to cope with his illness:

“I think the main theme was that I’m ok. It’s been a good life and I’m ok. Being a family man, yes, and now I know who I am. Yeah, because I couldn’t – when I woke up in the hospital I didn’t realize that no one could understand what I was saying… Yeah. it slowed me down but it keeps me busy. Closer to my family and my friends. Everyone’s been visiting and showing their support – oh yeah, well that was people that came from work and friends to see me in [hospital]. They always made me laugh.”

“I suppose I was looking forward to getting back to work and those sort of things, but not everything has happened. So, the dream is near and some of it’s gone further away. Some of it’s come closer.”

Finn described how his second and third songs captured his experience of overcoming his illness using his inner resources and celebrating the ‘end’ of his illness:

“… I was trying to explain exactly that this is what I have been through and I’ve come out the other side. It hasn’t got to the part of coming out the other side but how (my illness) affected my life and that I wasn’t very happy about having it but there you go, I had to grin and bear it and suffer on, see how we get through…because it [the song] was a celebration that everything has finished and it’s all back on the motorbike and we’re hunky dory again everything is back to normalish.”

Matthew reflected on his recovery, and the process of exploring and identifying internal and external coping strategies to overcome challenges in Songs 2 and 3:

“It is about coming home and all the changes that you were going through… I think I tried to say how grateful I was, and how I came at this stage and how far I wanted to take it. The people that helped me, along the way. Which is my wife, and my grandkids, and my kids.”

“Looking for the future. Like I said before, when I was in hospital my son says you can’t go to sleep, or whatever. You can’t die, because you’ve got another grandkid coming up. Now I’ve got another one, I’m looking forward to, in about 3 months’ time. So always you’ve got something to look forward to, in life. My ambition is now to grow old with my wife.”

Billy talked about how his second song contained the main message of his inner struggles in facing the physical changes and exploring inner resources associated with his injury:

“It’s sort of a play on words because moving in the moment, well, I couldn’t really walk through the motion of movement through the moment, if that makes sense… I literally was sitting still, because I was in my day chair and I couldn’t really walk away from the table. There was so much happening around me with turmoils of emotions and thoughts, my mum and dad’s emotions and thoughts… Sitting still in the middle of the mayhem, not running, not walking, but moving in the moment. And I realized I was still running the race, and I was still walking out my faith, even though I couldn’t walk.”

Billy went on to reflect on how his song described the importance of spirituality to get him through rehabilitation:

“Obviously my feet can’t walk at the moment, well at that time I couldn’t walk – but I’m still following my faith, I’m still following what I believe. Walking within my hope, taking steps in faith. Then I ride along for now – it’s like, I ride in a wheelchair for now, I’ll ride this wave for now, I’ll ride this rollercoaster, sit in the middle of mayhem and pray… I think it’s [faith, spiritual self] grown, it’s sustained me a lot, but it’s also grown a lot. Even people back home say you’ve just grown so much and, you know, I don’t necessarily see that myself until I reflect on it.”

“But, you know, looking back on these words, you know, I’ve just gone wow, my faith has really supported me in these times. You know, just being able to write these songs, was like, really helped me express where I was and reflect where I was.

### Recovery Journey 4: I Don’t Yet Have the Answers

One out of fifteen participants (David) described how his songs reflected on his work and home life and evaluating his values pre-injury, processing the changes following the accident and negotiating the discrepancies associated with his past and current self:

“It just meant that, a few beers and a smoke after work… I probably drunk a bit too much before I hurt myself…I used to be away during the week, so on the weekends I’d just go and mow the lawns and that, have a few beers in the shed and have a game of pool with the kids or whatever. Family time or – mm, and mucking around with old cars or something.”

He talked about how his second song reflected on his cognitive and emotional processes including the challenges and expectations associated with his illness:

“It’s how life has changed after the accident. Like I said in the song, life’s meant to get – not so hard, get easy, but it hasn’t. It hasn’t got any better, I don’t think anyway. Well, I couldn’t do nothing for myself and anything else. I still feel a bit like that now. Yeah, to have people helping me and all that. That side of it hasn’t really changed much. Felt like a burden stuck in this chair… Yeah, I’ve even lost that, just sitting around wondering what I’m going to do with myself. Yeah, I was better then because I thought I was going to get a little bit better. Now I’m just stuck with what I’ve got. I wrote some of these songs when I was sort of – gaining momentum. Then all of a sudden it hits you, and so, like this is it. This is all, it’s it… Yeah, I got to the plateau and then I fell off it.”

David reflected on how his third song captured his anxiety and fear about the uncertainty of his future:

“I was worried about going home and how I felt I was a burden and all that. In the end, it worked out to be right, didn’t it? Feeling like it would be a bit hard to transition back into family life… and how it was going to affect the kids and all that when I did get home. It affected them more than what we really know… Well, it did, didn’t it? I’m not in the family life anymore. Yeah, and it worked out the way I thought it would in the end… Yeah, and all of my fears come true. It still is challenging now.”

## Discussion

This study explored the experiences and meaning making of individuals in early rehabilitation who participated in a targeted identity-focused songwriting intervention. The four distinct recovery journeys that we identified illustrate that evolution of self-concept post-injury is complex but that with the exception of the one participant whose journey was characterized by a continuous struggle (journey 4, David), the therapeutic songwriting process enabled our participants to explore their self-concept, grief process, and reconceptualise their future selves in unique but useful ways. By exploring their perspectives of past, present, and future through song, our participants were able to recognize that the recovery journey enabled them to have a positive shift in sense of self (journey 1), negotiate inner conflicts and identify internal resources (journey 2), or explore potential ways to cope in the future (journey 3). These different journeys occurred despite the intervention protocol being delivered in the same way to all participants. This suggests that the process of exploring the past, present and future self through the songwriting process may have important value for people with acquired neurological disability, even if the effects of the intervention and the recovery journeys encountered by participants differ. We found that participants were able to negogiate discrepancies in identity continuity as they reconnected with their values, reflected on their relationships with others, family roles and social roles, while simultaneously confronting functional impairments and limitations (Ylvisaker and Feeney, 2000). This was all achieved through the creation of songs about their past, present and future. In addition, current findings appear to align with the post-traumatic growth literature, which suggests that meaning and social support are associated with positive psychological growth in individuals following life threatening events such as neurological injury/illness (Zeligman et al., 2018) and that focusing on one’s values may facilitate positive outcomes and psychological adjustment following TBI (Pais et al., 2017).

With respect to the participant in journey 4, it seems that despite the intervention, he was still in the midst of negotiating discrepancies in his self-concept and grappling with adjusting to a changed self as he confronted the impact of significant disability. Perhaps for this participant, the length of the intervention period was too short. Maybe he needed more time and space between sessions to process his explorations of self or more space between the creation of each song before he was ready to move further in his recovery journey. Alternatively, perhaps 12 sessions to create 3 songs was insufficient and this participant needed more sessions to support him through his journey, which is evidently slower than others. This may be especially apparent given that short-term memory and cognitive challenges may inhibit those with neurological disability, especially ABI, moving forward in a psychological process (Tamplin et al., 2016). This participant could have had limited cognitive resources and/or was experiencing psychological resistance, thereby requiring more time to process the meaning on his injuries in the context of his present and future life that resulted from his injury.

Another potential explanation for this participant’s more negative journey is that it might not have been the right time in his recovery process to participate in this personally confronting process. When experiencing such a crisis, it may be difficult to voice your thoughts as once you label your feelings and shape them into lyrics, you need to own them (Baker, 2013). It is possible that this participant had so much to deal with, including staying focused on his rehabilitation program, that reflecting on the present and contemplating the future was just too confronting for him at this time post-injury. Timing of intervention implementation is an important consideration in music therapy practice. Being able to determine “when is it too early? when is it too late? and when will the intervention have the biggest impact?” are important questions research should seek to answer. Further research focused specifically on the timing of therapeutic songwriting implementation is needed to further develop our knowledge and ensure best practice.

### Study Limitations

This study reported on qualitative analyses of interview data from participants who were available and willing to be interviewed at 6 months post-intervention. It is possible that those who were not able to be followed up, intentionally did not respond to our invitations to be interviewed because their experiences were not as positively transformative. If so, we may have an inherently biased sample of participants who were willing to share their experiences. If we had studied carefully the journeys reported in all of the participants’ songs, we may have encountered more participants who experienced journey 4 or experienced other less transformative journeys than those reported by our participants. Considering the quantitative data that suggests some participants experienced positive changes in identity while others had negative changes (Roddy et al., 2017, 2018), these findings need to be viewed with caution and not necessarily as representative of all people’s recovery journeys. Further investigation into the journeys that were negative is warranted to gain a deeper understanding of how songwriting influences recovery and the reconstruction of the self-concept.

### Implications of Study and Recommendations for Future Research

Our research indicates that a therapeutic songwriting protocol that specifically targets an exploration of the self-concept facilitates a recovery journey, of one form or another. The focused process of exploring the self through a songwriting experience enables people with neurological disability to reflect on their thoughts, and through the crafting of lyrics and music, revisit their perspectives over and over again (Baker, 2015). This revisiting that occurs when re-reading and refining their lyrics (Tamplin et al., 2016), fosters opportunities to reframe feelings about their situation with the aim of enabling people with neurological disabilities to reach a consensus on what is meaningful in life and resolve potential conflicts between what was, is, and can be. These findings are likely to have clinical implications for music therapy clinicians, as recognizing transformative moments that match one of the four possible recovery journeys might inform whether further exploration is needed (and the issues subsequently included in song lyrics). This knowledge also gives clinicians permission to allow people to experience conflict, knowing that conflict is integral to some people’s recovery journey. Clients may be inclined to drop out of treatment when issues being explored become confronting. However, clinicians can use findings from this study to share with their clients, informing them that conflict? may be an integral part of the journey to a healthier and more positive self-concept and more positive view of their future.

We have a long-standing commitment to using therapeutic songwriting with vulnerable populations because of the potential for this medium to tell people’s stories in a form (verbal) with which most people are familiar. Music in this case can be used to convey mixed or ambivalent emotions or to further intensify the meaning of the lyrics. However, we acknowledge that the more traditional music therapy method of improvisation also has the potential to explore people’s feelings and sense of self. Future research could explore and compare aspects of the music therapy process by improvising on the referential theme of past self, then on present self, and then on an imagined future self and comparing the journey and outcomes with those that use songwriting which uses verbal processing.

Using therapeutic songwriting as a tool to address a disintegrated or negative self-concept and identity is emerging as an area of increasing importance across a broad range of clinical areas (Baker and MacDonald, 2017). Identity crises may be experienced by people who have experienced abuse, displacement, mental illness, or recent diagnoses of terminal illnesses, all of whom might benefit from a tailored songwriting program, such as that used in the current study, which specifically focuses on reflecting on past, present, and future. Clinicians are encouraged to consider the broader applicability of this research and whether their clients with identity crises of differing origin respond to the songwriting protocol in ways that map onto the journeys presented here.

## Conclusion

Our study found that participants with a SCI or ABI were able to constructively use a therapeutic songwriting process to reflect on and explore aspects of their pre-injured self, present self, and an imagined a future self as someone living with a permanent acquired disability. By synthesizing the participants’ own reflections on their songs’ meanings and comparing these perspectives with the lyrical content of the songs, we were able to synthesize the experiences of 15 participants into 4 distinct recovery journeys. Our research shows that through the creation of three personally meaningful songs, participants with acquired neurological disabilities have the opportunity to reconceptualise what is valuable to them, to recognize and utilize their inner resources, to confirm their values, and to identify coping strategies that will support them as they contemplate a future with permanent disability. Songwriting was found to be a powerful means to explore the self and engage in a recovery journey.

## Author Contributions

FB was responsible for the overarching design of the study and design of the intervention. Y-EL and JT conducted the songwriting interventions. FB and Y-EL undertook the interpretative phenomenological analysis and then sought further input from the remaining authors. All authors contributed to the development of the research questions and interview questions presented to participants and writing of the manuscript.

## Conflict of Interest Statement

The authors declare that the research was conducted in the absence of any commercial or financial relationships that could be construed as a potential conflict of interest.
